# Self-Assembled, Adjuvant/Antigen-Based Nanovaccine Mediates Anti-Tumor Immune Response against Melanoma Tumor

**DOI:** 10.3390/polym10101063

**Published:** 2018-09-25

**Authors:** Santhosh Kalash Rajendrakumar, Adityanarayan Mohapatra, Bijay Singh, Vishnu Revuri, Yong-Kyu Lee, Chang Seong Kim, Chong-Su Cho, In-Kyu Park

**Affiliations:** 1 Department of Biomedical Science and BK21 PLUS Center for Creative Biomedical Scientists at Chonnam National University, Chonnam National University Medical School, Gwangju 501-746, Korea; kalash1288@gmail.com (S.K.R.); mkaditya55@gmail.com (A.M.); 2 Research Institute for Bioscience and Biotechnology, Nakkhu-4, Lalitpur 44600, Nepal; singhbijay@ribb.org.np; 3 Department of Green Bioengineering, Korea National University of Transportation, Chungju 27469, Korea; vishnurevuri91@gmail.com (V.R.); yklee09@naver.com (Y.-K.L.); 4 Department of Internal Medicine, Chonnam National University Medical School, 42 Jebongro, Gwangju 501-757, Korea; laminion@hanmail.net; 5 Department of Agricultural Biotechnology and Research Institute for Agriculture and Life Sciences, Seoul National University, Seoul 08826, Korea; chocs@snu.ac.kr

**Keywords:** poly I:C, adjuvant, antigen, melanoma, polyethylenimine, immunotherapy

## Abstract

Malignant melanoma is a highly aggressive type of cancer that requires radical treatment strategies to inhibit the cancer cell progression and metastasis. In recent years, preclinical research and clinical trials on melanoma treatment have been considerably focused on the adjuvant-based immunotherapy for enhancing the immune response of innate immune cells against cancer cells. However, the clinical outcome of these adjuvant-based treatments is inadequate due to an improper delivery system for these immune activators to reach the target site. Hence, we developed a vaccine formulation containing tumor lysate protein (TL) and poly I:C (PIC) complexed with positively charged poly (sorbitol-*co*-polyethylenimine (PEI) (PSPEI). The resulting ionic PSPEI-polyplexed antigen/adjuvant (PAA) (PSPEI-PAA) nanocomplexes were stable at the physiological condition, are non-toxic, and have enhanced intracellular uptake of antigen and adjuvant in immature dendritic cells leading to dendritic cell maturation. In the murine B16F10 tumor xenograft model, PSPEI-PAA nanocomplexes significantly suppressed tumor growth and did not exhibit any noticeable sign of toxicity. The level of matured dendritic cells (CD80+/CD86+ cells) in the tumor draining lymph node of PSPEI-PAA treated tumor mice were enhanced and therefore CD8+ T cells infiltration in the tumor were enriched. Additionally, the cytotoxic T lymphocytes (CTLs) assay involving co-culturing of splenocytes isolated from the PSPEI-PAA-treated mice with that of B16F10 cells significantly revealed enhanced cancer killing by the TL-reactivated CTLs compared to untreated control mice bearing tumor. Therefore, we strongly believe that PSPEI-PAA nanocomplexes could be an efficient antigen/adjuvant delivery system and enhance the antitumor immune response against melanoma tumor in the future clinical trials.

## 1. Introduction

According to the 2018 cancer statistics by American Cancer Society, the overall estimated new cases and death rate in melanoma accounts for 5.2% of the total cancer cases [1]. In current clinical trials, cancer immunotherapy has been a rapidly evolving strategy for the treatment of late stage melanoma cancer [2,3,4]. The immunotherapy-based treatment strategies such as blocking immune checkpoints using PDL-1, PD-1 or CTLA-4 antibodies, adoptive T cell transfers, adjuvants-antigen based vaccine, and dendritic cell-based therapy have great potential in enhancing anti-tumor immune responses specifically against the cancer cells [5,6,7]. Although the recent preclinical studies were more focused on the activation of innate immunity using antigen and adjuvants against the cancer [8,9,10], antigen/adjuvant-based immunotherapy has provided more specific killing of tumor cells, reduced side effects and prevented tumor recursion [11,12]. However, the major challenge to antigen/adjuvant-based vaccines are limited accumulation of the administered vaccine in the lymph node, their reduced uptake by the antigen presenting cells and poor immunogenicity [13]. Hence, an efficient delivery system becomes essential for transporting antigen/adjuvant agents to the target site in order to enhance the anti-cancer immune response and provide better anti-cancer effects.

Nanoparticle based immunotherapy has emerged along with the development and discovery of novel adjuvants and antigens [14]. Nanovaccines are exceedingly capable of initiating antitumor immune response, thereby preventing cancer cell evasion as well as metastasis [15]. Here, the co-delivery of antigen and adjuvants in the nanoparticle form promoted maturation and activation of antigen presenting cells, which in turn led to activation of T lymphocytes for killing of cancer cells [16,17]. The activated T lymphocytes could be differentiated further into memory T cells for withholding the proliferation and metastasis of cancer cells to the other organ sites [18]. By utilizing polymeric nanoparticles, proper packaging of the antigenic proteins and adjuvants can be achieved more efficiently for successful delivery to antigen presenting cells like dendritic cells [14,19]. Effective delivery of antigen/adjuvant by the nanoparticles has enhanced the maturation of antigen presenting cells and thus further improved the anti-cancer immune response against cancer cells [14,19]. Polysorbitol based transporters are osmotically active gene transporters used for delivering genes to the cancer cells. Recently Firdous J. et al. has also tried to use PSPEI polymer polyplexed with respiratory syncytial virus glycoprotein (RGp) antigen for long term immunity [20,21].

Among all the adjuvant compounds, Toll-like receptors (TLRs) agonists like poly I:C and imiquimod have been widely studied due to its potential ability to boost anti-tumor immune response in cancer immunotherapy [22,23]. Synthetic double-stranded RNA poly I:C (PIC), an TLR-3 agonist, has been known to exhibit anti-cancer activity and activate the pathway responsible for the expression of pro-inflammatory cytokine type I IFNα as well [24,25,26]. In addition, PIC acts as a potent DC maturation agent and induces a Th1 immune response against antigens specific to the cancer cells [27,28]. For an antigen based anti-cancer immune response, novel synthetics peptides and melanoma-associated antigens have been used for activating cytotoxic T cells (CTLs) to kills cancers [29,30,31]. However, it has been well known that the use of tumor protein lysate facilitates a more robust immune response against multiple unique antigenic determinants in tumors and reduces the immune escape of cancer cells [32]. In the current study, we formulated nanocomplexes by using PSPEI polymer polyplexed with PIC and lysate protein (PSPEI-PAA) from B16F10 cancer cell line for the antigen/adjuvant-based immunotherapy against the melanoma tumor model.

## 2. Materials and Methods

### 2.1. Materials

Poly I:C, Ovalbumin (OVA), and linear PEI 423 MW were purchased from Sigma Aldrich (St. Louis, MO, USA). Sorbitol diacrylate (SDA) was bought from Monomer-Polymer & Dajac Labs (Trevose, PA, USA). GM-CSF, IL-4 recombinant protein and all the fluorescent labelled antibodies for flow cytometry was obtained from Thermo Fisher Scientific Korea Ltd. (Seoul, Korea).

### 2.2. Synthesis of PSPEI

As per the previous reference [33], the synthesis of PSPEI was performed by Michael addition reaction. Briefly, linear PEI (MW 423) and SDA (MW 290.27) were dissolved in DMSO separately and later while stirring, SDA was slowly added to PEI solution at a 1:1 molar ratio. With continuous stirring, the mixture was incubated at 80 °C for 24 h. The product was dialyzed (MWCO: 3500) and lyophilized for further use.

### 2.3. Tumor Cell Lysate

1 × 10^8^ B16F10 cells were trypsinized and washed with phosphate-buffered saline (PBS) buffer. Four cycles of freeze-thaw method were followed using liquid N_2_ and 37 °C water bath. After a brief sonication, complete cell death was confirmed using trypan blue staining and later centrifuged at 10,000× RPM for 10 min to obtain the protein lysate. The protein lysate was filtered through Amicon Ultra-15 Centrifugal Filter (Merck, Kenilworth, NY, USA). The protein lysate was lyophilized and stored as powder in 4 °C.

### 2.4. Formulation of PSPEI Complexed PIC and TCL (PSPEI-PAA)

All the components were dissolved in PBS (pH 7.4). First, the PSPEI-PIC polyplex were prepared by mixing PSPEI and PIC at appropriate ratio (50:1) and incubating for 15 min. Later, different amounts of lysate protein and polyplex were mixed and incubated for 15 min.

### 2.5. Characterization of PSPEI-PAA

The size and zeta potential of the PSPEI-PAA nanocomplex was measured in Zetasizer Nano ZS (Malvern, Malvern, Worcs, UK). The condensation of PIC by PSPEI was assessed by running the samples in 1% agarose gel electrophoresis and red fast staining was performed in order to visualize under a gel documentation system.

### 2.6. Scanning Electron Microscope Imaging

For HR-SEM analysis, 250 µL of 400 µg/mL PSI was added to 250 µL of 8 µg/mL poly I:C and incubated at room temperature for 15 min. Later 500 µL of 100 µg/mL OVA was added to the above complex and further incubated at room temperature for 15 min. Finally, 10 µL of the above sample was added on the silica grid, dried, sputter coated with platinum and further used for HR-SEM analysis (High Resolution Field Emission Scanning Electron Microscope JSM-7610F, JEOL Ltd., Akishima, Tokyo, Japan).

### 2.7. Isolation of Bone Marrow Derived Dendritic Cells (BMDCs)

Bone marrow cells are harvested from the 8 weeks old mice’s femur and tibia bone and cultured in complete dendritic cell (DC) media containing Roswell Park Memorial Institute (RPMI) 1640 media along with 10% fetal bovine serum (FBS)/1% antibiotic, 10 ng/mL GM-CSF, and 10 ng/mL IL-4. Media change were done on the 3rd and 6th day and finally on the 7th day, immature dendritic cells were obtained and confirmed with flow cytometry using CD11c, MHCII, and CD80 antibodies.

### 2.8. In Vitro Cell Viability Assay Using MTS Reagent

In vitro cell cytotoxicity in the RAW264.7 cell line and in DC2.4 cell lines was evaluated by MTS cell viability kit. 1 × 10^4^ cells/well were seeded onto 96-well plate and incubated overnight in the culture medium. The medium was replaced with samples in 100 μL of RPMI 1640 medium and incubated for 24 h and cell viability was assessed by addition of 3-(4,5-Dimethylthiazol-2-yl)-5-(3-carboxymethoxyphenyl)-2-(4-sulfophenyl)-2H-tetrazolium, inner salt (MTS) reagent and absorbance reading was taken at 490 nm after 4 h. Similarly, for the BMDCs, cells treated with samples at different weight ratios were incubated for 24 h, and the cell viability was evaluated similarly to the previously described method.

### 2.9. Confocal Microscope Imaging

To determine the intracellular internalization of PSPEI-PAA nanocomplex, OVA albumin- Fluorescein isothiocyanate (FITC) was used a model antigen in the PSPEI-PAA nanocomplex. iDCs cells were seeded at 1 × 10^4^ cells per well in Lab-Tek^®^ Chamber Slides (Thermo Scientific, Waltham, MA, USA) and incubated overnight at 37 °C and 5% CO_2_. PSPEI-PAA nanocomplex at different weight ratios in OPTI-MEM^®^ serum free media were incubated with the cells for 4 h, and after a brief wash with 1X PBS, the cells were incubated with 4% Paraformaldehyde (PFA). Nuclear staining was performed using 4′,6-diamidino-2-phenylindole (DAPI) in gold anti-fade reagent. The fluorescence in the cells were visualized using a confocal laser scanning microscope (CLSM).

### 2.10. Fluorescence-Activated Cell Sorter (FACS) Analysis

1 × 10^6^ BMDCs were cultured overnight in 6 well plate containing complete dendritic cell media in 5% CO_2_ incubator at 37 °C. Then, PSPEI-PAA at different weight ratio were prepared in serum free media. The treatment period was 4 h and after that, cells were washed in PBS twice and stained with CD11c-APC antibody for 30 min. Later, the cells were fixed with 1% formaldehyde for 15 min. Flow cytometry analysis was performed in BD FACSCalibur (BDbiosciences, San Jose, CA, USA). The data was analyzed and plotted using winMDI 2.8 software (ver.2.8, The Scripps Institute, La Jolla, CA, USA).

### 2.11. B1610 Tumor Model, Animal Grouping and Immunization

According to the institutional guidelines of the Chonnam National University Medical School and Chonnam National University Hospital (CNU IACUC-H-2015-47), Korea, all experiments involving live animals were performed. Female BALB/c nude mice (6–8 weeks old) were purchased from Orient Bio Inc., Seongnam-si, Korea. 5 × 10^5^ B16F10 cells were subcutaneously injected into the right flank of the mice and later mice bearing 100 mm tumor volume were used for anti-tumor studies. Here, the tumor volume = (tumor length) × (tumor width)^2^/2. For immunization, mice were randomly divided into five groups (n = 6). Samples were injected peritumoral consequently for 4 days, with PBS alone as control.

### 2.12. Isolation of Immune Cells from Primary Tumor and Tumor Draining Lymph Node

Tumor tissue were cut into less than 3 mm pieces and incubated in 5 mL of dissociation solution containing 100 μg/mL DNase, 170 µg/mL collagenase type I, and type IV for 30 min at 37 °C. Digested tissue were passed into 40 μm cell strainer and then washed twice by centrifugation at 2000 rpm for 10 min. ACK lysis buffer was used to lysis the red blood cells. The cells were counted and stained with CD3, CD4, FOXP3 and CD8 antibodies for flow cytometry analysis. Similarly, cells from lymph node are isolated by mechanically digesting the lymph node tissue and then washed twice by centrifugation at 2000 rpm for 10 min. The isolated cells were stained with CD11c, CD80 and CD86 antibodies for flow cytometry analysis.

### 2.13. Splenocyte Proliferation Assay

Splenocytes were isolated from the spleen excised from the treatment mice using cell strainer. The cells were cultured in IL-2 (25 IU/mL) and B16F10 cell lysate protein (1 μg/mL) for 3 days. Before the co-culture, B16F10 cells seeded (1 × 10^4^ cells/well) in 96 well plate were treated with 30 μg/mL of mitomycin C for 3 h. After a brief wash, the splenocytes were added to B16F10 cells at 25:1, 50:1 and 100:1 ratio and incubated for 6 h at 37 °C in CO_2_ incubator. Lactate dehydrogenase (LDH) assay was performed using the supernatant obtained from each treatment.

### 2.14. Statistical Analysis

Statistical analyses were performed using GraphPad Prism 5 (ver. 5, Graphpad, La Jolla, CA, USA). Graphical data are expressed as the average mean ± SEM (standard error of the mean). Two-way ANOVA was used to compare different treatment groups. Differences were considered significant at * *p* ≤ 0.05, ** *p* ≤ 0.01, and *** *p* ≤ 0.001.

## 3. Results

### 3.1. Physiochemical Characterization of Polysorbital-Polyethylenimine (PSPEI) Complexed with Poly I:C (PIC) and Tumor Lysate (TL) (PSPEI-PAA) Nanocomplex

**Figure 1:**
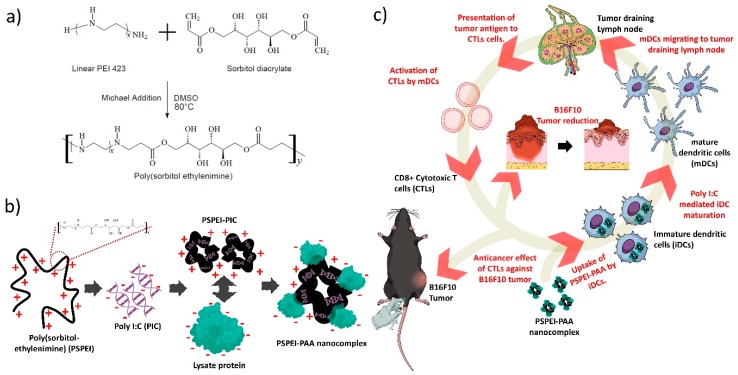
PSPEI-PAA nano-vaccine mediates anti-tumor immune response against melanoma tumor. (**a**) Schematic representation of PSPEI synthesized by Michael addition method, (**b**) pictorial representation of formulation of PSPEI-PAA nanocomplex using PSPEI polymer complexed with poly I:C and lysate protein in sequential manner, and (**c**) PSPEI-PAA administration in B16F10 tumor via peritumoral route initiates the antitumor immune response by maturating dendritic cells and in turn activating the cytotoxic T cells. The activated CTLs kills the cancer cells, therefore leading to reduction in the tumor burden.

**Figure 2:**
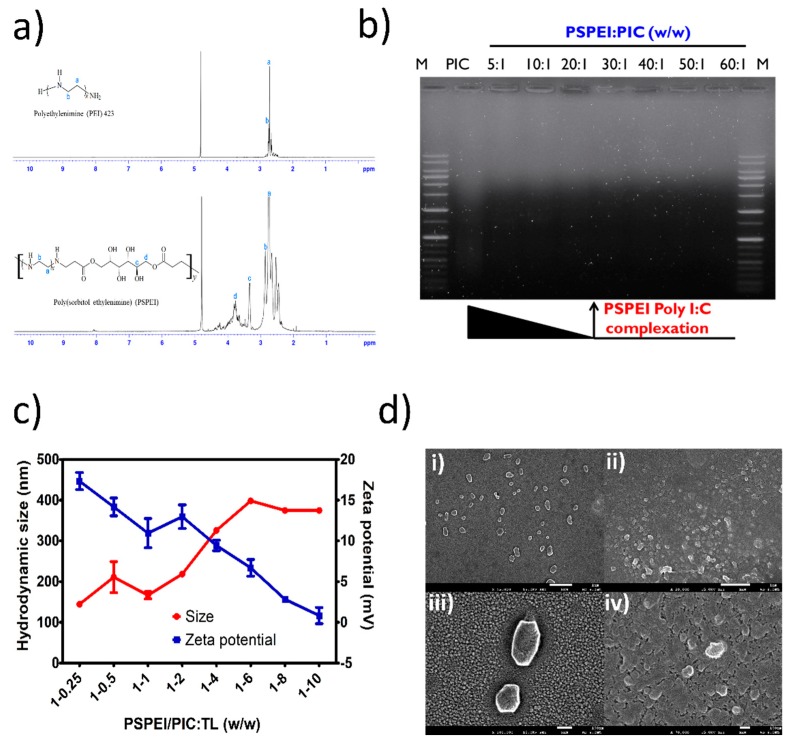
Characterization of PSPEI-PAA nanocomplex. (**a**) ^1^H NMR of PEI435 and PSPEI; (**b**) gel retardation assay of PSPEI complexed with PIC at different weight ratio; (**c**) hydrodynamic size and zeta potential of PSPEI-PAA nanocomplex at different weight ratios of PSPEI/PIC to TL and (**d**) SEM image of (i and iii) PSPEI-PIC and (ii and iv) PSPEI-PAA nanocomplex.

As shown in Figure 1a, PSPEI polymer was synthesized by Micheal addition reaction between SDA and LMW PEI. A ^1^H NMR spectrum with the corresponding proton groups of PEI and PSPEI was shown in Figure 2a. The distinct peaks of protons were visible in the NMR spectra of PEI and PSPEI and the characteristic proton groups of the PSPEI have been assigned. As shown in the Figure 2a, the proton peaks of SDA ranged from 3.4–4.1 ppm appeared after the synthesis of PSPEI confirming the successful reaction between sorbitol and the amine groups of PEI. Polysorbitol-*co*-polyethylenimine polymer are efficient gene carriers due to their positive surface charge enabling them to condense plasmid DNA and siRNA more efficiently [33,34]. Hence, we assessed the condensation of PIC with PSPEI polymer through gel retardation assay. In Figure 2b, the complete condensation of PIC with PSPEI was clearly noticed at the PSPEI: PIC ratio higher than 20:1 (*w*/*w*). Schaffert et al. has previously shown that the linear PEI are more efficient in condensing PIC as well as superior delivery efficiency compared to branched PEI [35]. Since the PSPEI/PIC polyplex has retained positive surface, we further complexed it using protein lysate at a different weight ratio. The cell lysate protein isolated from B16F10 cells through heat shock method has mostly negative surface at pH 7.4. As the amount of lysate protein increased, the size of the PSPEI-PAA complex has been increased with reduction in zeta potential, respectively (Figure 2c). Therefore, it suggests that PSPEI/PIC polyplex positive surface charge was compromised with binding of negatively charged lysate protein. Syga et al., has previously shown that albumin protein and plasmid DNA added sequentially to the PEI polymer has compact and stable polyion complex as well as improved unpacking of the cargo inside the cells [36]. Hence, we prepared this PSPEI-PAA nanocomplex through sequentially mixing process for better stability of the nanocomplex and efficient delivery of the antigen/adjuvant inside the immune cells. The SEM analysis also shown that the size of the PSPEI-poly I:C polyplex was 164 nm (±11.76), whereas the size of PSPEI-PAA nanocomplex was 188 nm (±12.68) (Figure 2d).

### 3.2. Viability of Immune Cells Treated with PSPEI-PAA Nanocomplex

**Figure 3:**
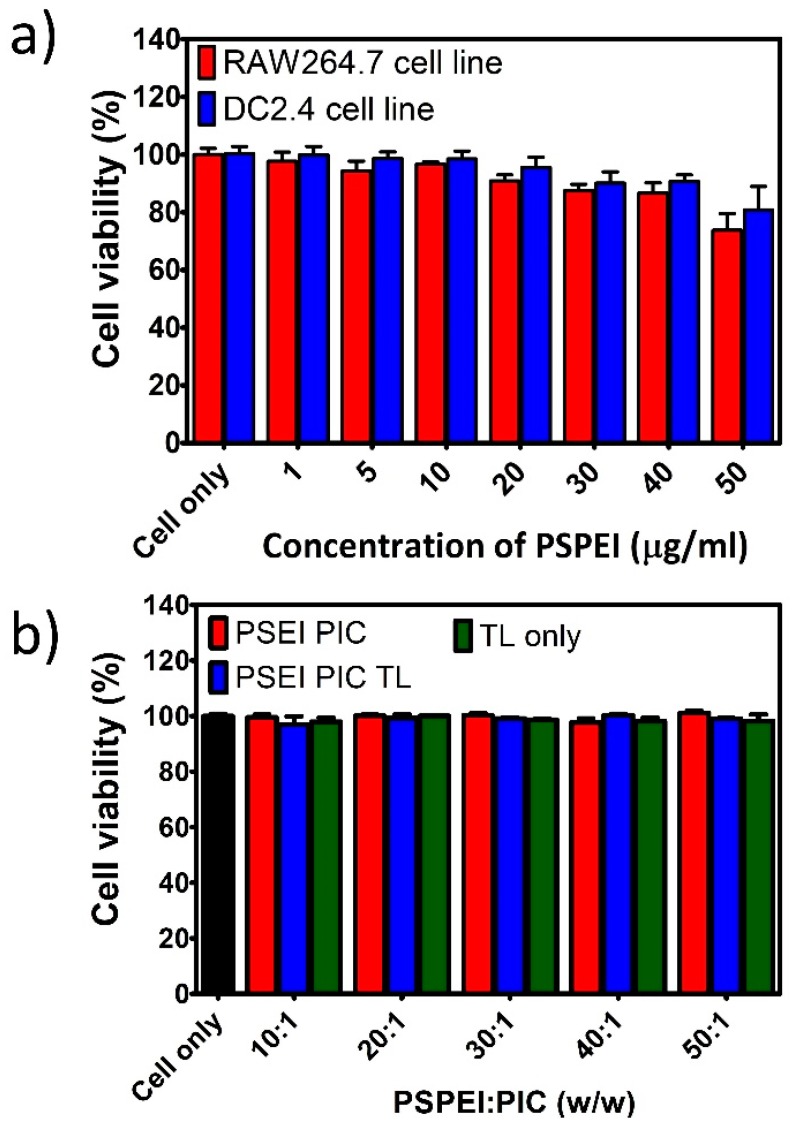
Cell viability of immune cells treated with PSPEI-PAA nanocomplex for 24 h. (**a**) Viability of RAW264.7 and DC2.4 cell line treated with PSPEI at different concentration and (**b**) viability of immature dendritic cells treated with PSPEI-PAA nanocomplex at different weight ratios of PSPEI and PIC. (n = 4, SEM).

The cell viability of immune cells treated with PSPEI polymer and the PSPEI-PAA nanocomplex was assessed by MTS assay. Initially, the cytotoxicity assessment of PSPEI polymer alone was analyzed in the immune cell line. In Figure 3a, RAW264.7 macrophage cell line and DC2.4 dendritic cell line showed no significant toxicity after treatment with PSPEI polymer for upto 50 μg/mL concentration. Later, the cell viability of the PSPEI-PAA nanocomplex was examined in the immature bone marrow derived dendritic cells (BMDCs). Here, the cells were treated with different weight ratio of PSPEI-PAA nanocomplex exhibited no sign of toxicity as shown in Figure 3b. As controls, PSPEI/PIC polyplex and lysate protein were used and they did not show any cytotoxic effect over the dendritic cells.

### 3.3. Intracellular Uptake of PSPEI-PAA Nanocomplex in Immature Dendritic Cells

**Figure 4:**
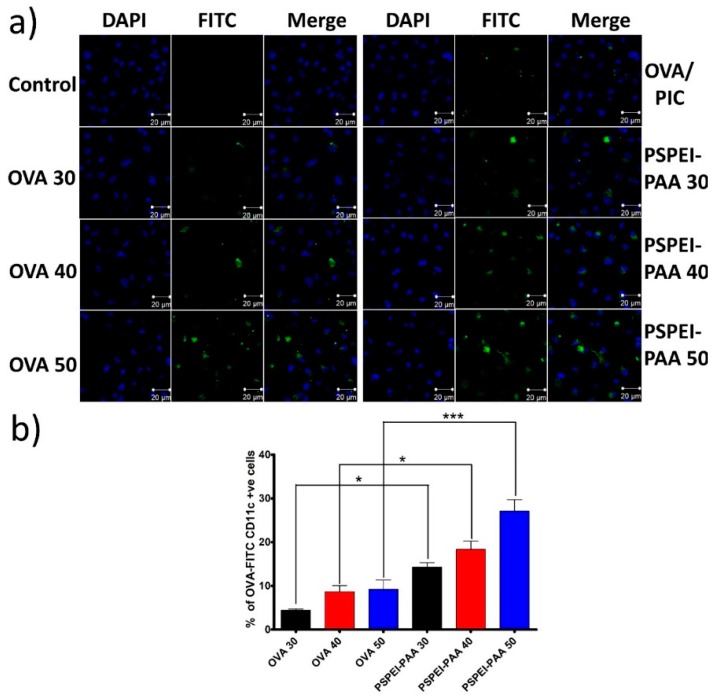
Intracellular uptake of PSPEI-PAA nanocomplex in immature BMDCs. (**a**) CLSM image of immature BMDCs treated with PSPEI-PAA containing FITC labelled OVA; (**b**) flow cytometry analysis of CD11c+ BMDCs internalized with OVA-FITC in PSPEI-PAA. (n = 4, SEM, * *p* ≤ 0.05, and *** *p* ≤ 0.001).

Before the assessment of intracellular uptake of PSPEI-PAA in immature BMDCs, OVA-FITC was prepared by conjugating amine group of OVA with isothiocynate group of FITC and confirmed the FITC content through standard plot using the absorbance of fluorescein (495 nm). Immature BMDCs were treated with PSPEI-PAA containing OVA-FITC as model antigen and imaged under CLSM. As seen in Figure 4a, the fluorescence intensity of FITC was found to be enhancing in the BMDCs with increase in PSPEI-PAA nanocomplex concentration, whereas the antigen alone showed less fluorescent intensity in the BMDCs. The fluorescence intensity of BMDCs treated with PSEI-PAA 50 (50:1 *w*/*w*, PSPEI:PIC) was higher than that of OVA 50. Through FACs analysis, it was confirmed that PSPEI-PAA has shown enhanced intracellular uptake in the BMDCs with respect to increase in the concentration, whereas OVA-FITC alone did not show any significant increase in the intracellular uptake (Figure 4b). This signifies that PSPEI-PAA nanocomplex enhances the intracellular uptake of antigen in the BMDCs compared to antigen alone.

### 3.4. Bone Marrow Derived Dendritic Cell Maturation by PSPEI-PAA Nanocomplex

**Figure 5:**
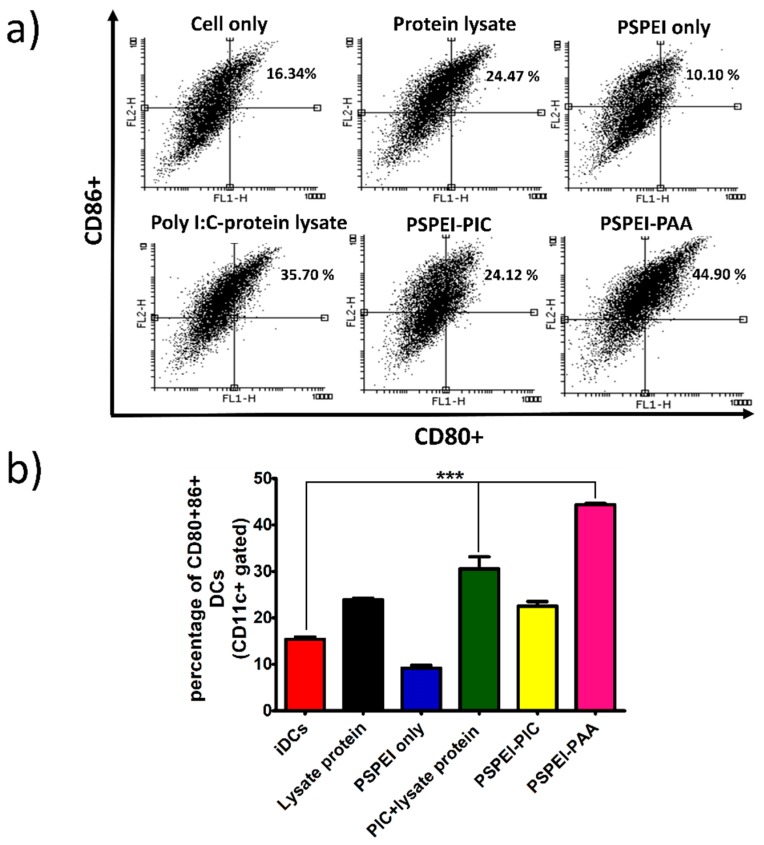
Characterization of bone marrow derived dendritic cells (BMDCs) using flow cytometry. (**a**) Flow cytometry analysis of BMDCs treated with PSPEI-PAA nanocomplex for 24 h and stained with maturation markers like CD80 and CD86 antibodies and (**b**) bar graph plot of CD80+CD86+ DCs (gated with CD11c+ cells). (n = 3, S.E.M, *** *p* < 0.001).

After assessing the intracellular uptake of PSPEI-PAA nanocomplex in the day 6 BMDCs, the BMDCs were treated with the PSPEI-PAA nanocomplex for 24 h and analyzed for DC maturation surface markers such as CD80 and CD86. Both PIC-lysate protein mixture and PSPEI-PAA treatments have shown significant increase in the expression of CD80/CD86 markers compared to the non-treated cells (Figure 5a). However, the PSPEI-PAA treated BMDCs have shown enhanced expression of CD80/86 markers compared to PIC-lysate protein mixture (Figure 5b). This signifies that the enhanced intracellular uptake of PSPEI-PAA nanocomplex has attributed towards the increase in the maturation of BMDCs.

### 3.5. Antitumor Activity of PSPEI-PAA Nanocomplex in B16F10 Tumor Model

**Figure 6:**
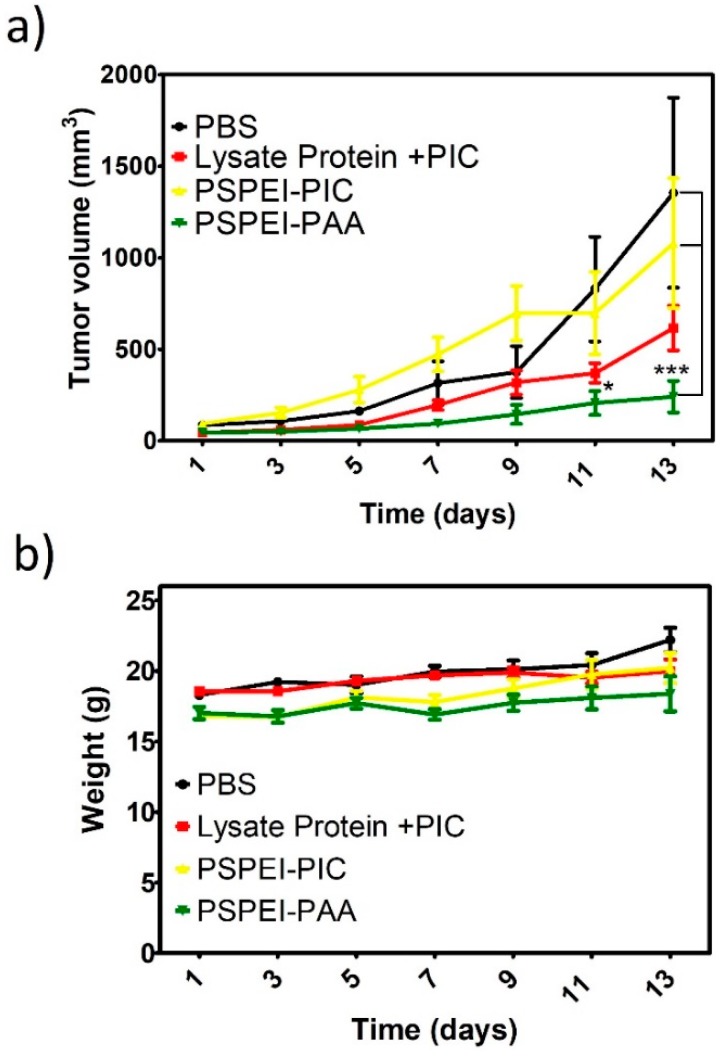
Antitumor effect of PSPEI-PSPEI-PAA in B16F10 tumor model. (**a**) Tumor volume, and (**b**) body weight of the treatment B16F10 tumor mice. (n = 4, SEM, * *p* ≤ 0.05, and *** *p* ≤ 0.001).

B16F10 subcutaenous tumor model was developed and samples were injected by peritumorally. The tumor mice were vaccinated for the initial four consecutive days and tumor volume was measured simultaneously for two weeks from the day of first treatment. In Figure 6a, shows that the tumor volume of PSPEI-PAA was significantly decreased than that of PBS control or PSPEI/PIC polyplex. Although the protein lysate mixed with PIC also showed reduced tumor volume, it was not as significant as the PSPEI-PAA treatment group. The treatment for all the groups showed no effect over the body weight of the mice (Figure 6b). This signifies the PSPEI-PAA has no side effects on the mice.

### 3.6. Characterization of Immune Cells from Tumor Draining Lymph Node and Tumor

**Figure 7:**
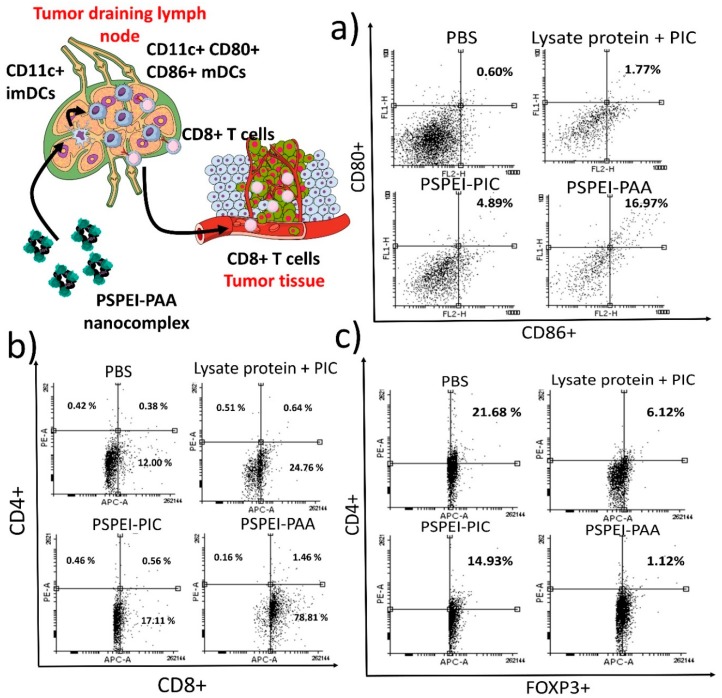
Characterization of dendritic cells and T lymphocytes in tumor draining lymph node and tumor tissues. (**a**) Dendritic cell maturation assessment of isolated cells from tumor draining lymph node and stained with CD80 and CD86 antibodies (gated with CD11c+ cells) and (**b**) assessment of CD4+ Th, CD8+ Tc and (**c**) FOXP3+ Treg cells in the tumor tissue (gated with CD3+ cells).

After vaccination of B1610 tumor mice with PSPEI-PAA nanocomplex, single cell suspension of the isolated tumor and tumor draining lymph node were then prepared for the assessment of the matured DCs, CD8+, CD4+ and CD4+FOXP3+ T lymphocytes. The characterization of lymph node cells from the PSPEI-PAA nancomplex treatment mice revealed that 16.97% (±5.92) of cells were CD80+/86+ DCs, whereas PIC-lysate protein mixture treatment group has 1.77% (±3.25) CD80+86+ DCs only (Figure 7a). This signifies that PSPEI-PAA treatment has enhanced the maturation of DCs in the tumor draining lymph node. Additionally, the level of CD3+CD8+ T lymphocytes infiltrated in the tumor were found to be 78.81% (±6.89) in the PSPEI-PAA nanocomplex treatment group, whereas in PIC-lysate protein treatment group has 24.76% (±4.32) only (Figure 7b). However, there was no significant change in the CD3+CD4+ T helper cell population in all the treatment groups. Additionally, the level of CD3+CD4+FOXP3+ Treg cells in the tumor of PSPEI-PAA nanocomplex treatment group were declined compared to the PBS control group (Figure 7c). Overall, the maturation of DCs by PSPEI-PAA nanocomplex has activated the CD3+CD8+ cytotoxic T cells for the tumor killing and due to unknown reasons the CD3+CD4+FOXP3+ Treg cell population were affected by the PSPEI-PAA nanocomplex administration in the B16F10 tumor mice.

### 3.7. Antitumor Immune Response of PSPEI-PAA Nanocomplex Activated Splenocytes

**Figure 8:**
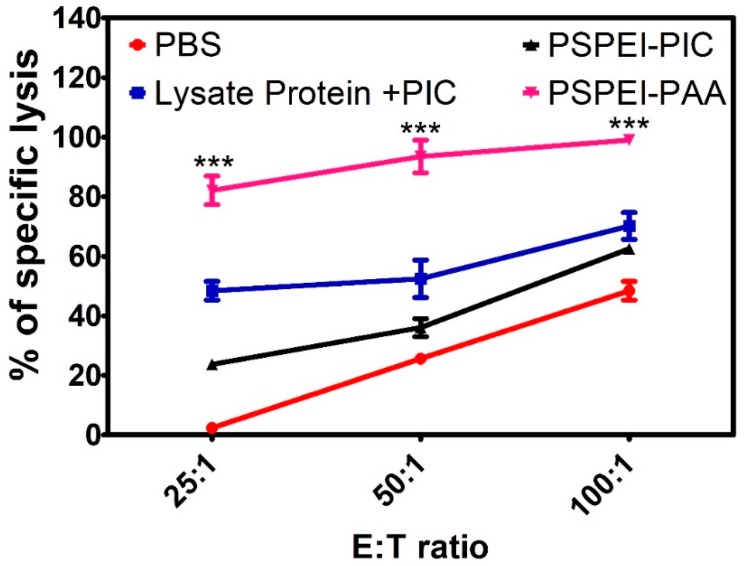
LDH assay of splenocytes cocultured with Mitomycin C treated B16F10 cancer cells. (n = 4, SEM, *** *p* ≤ 0.001).

Splenocytes were isolated from the spleen obtained from the treatment groups and were cocultured with B16F10 cells for 6 h and LDH assay was performed to assess the cell-mediated cytotoxicity. In Figure 8, shows that the PSPEI-PAA showed enhanced lysis of the B16F10 cells compared to the controls. This signifies that cytotoxic T cells in the splenocytes population in the PSPEI-PAA were showing more of a killing effect towards B16F10 cells and were also responsible for the anti-tumor immune response in the tumor mice.

## 4. Discussion

For immunotherapy against melanoma, a nanoparticles-based immunomodulatory agent has been designed and engineered for antigen-specific immune responses acting against melanoma tumor. According to the previous study [33], polysorbitol-*co*-polyethylenimine (PSPEI) polymer was synthesized by Michael-addition reaction between SDA and LMW PEI (*M*_W_ 423) [37]. Due to the highly positively charge, PSPEI has condensed PIC more efficiently and it was confirmed through gel retardation assay that PSPEI formed stable complex with PIC at 20:1 (*w*/*w*) (Figure 2b). Later, PSPEI-PIC polyplex was complexed with lysate protein at different weight ratios and it was observed that the PSPEI-PIC polyplex hydrodynamic size has increased proportionally with the addition of protein lysate while the surface charge of the PSPEI-PIC polyplex has reduced with an increase in protein lysate concentration (Figure 2c). Generally, polyethylenimine-based nanoparticle tends to aggregate with the protein molecules and form a large size [38]. Similarly, here the negatively charged lysate protein in excess amount has attributed to aggregation and reduced surface charge of the PSPEI-PAA nanocomplex, hence optimal ratio at 1:1 (*w*/*w*) was considered for the further experiment. Cationic polymeric nanoparticles are promising carriers for delivering biomacromolecules such as DNA, proteins or the combination of both to the target site, since it can load multi-cargo through electrostatic interaction and carry them to the target site more efficiently [39]. Therefore, PSPEI-PAA nanocomplex was further studied to understand its effects over the immune cells required for inducing anti-cancer immune response.

Commonly, branched PEI (bPEI) are considered to be highly toxic to the cells due to its high positive charge density [40]. However, the PSPEI polymer has been prepared using LMW linear PEI and also has comparatively lower surface charge than bPEI, hence it could be assumed that the cytotoxic effect over the immune cells could be minimal. Henceforth, macrophage RAW264.7 and dendritic DC2.4 cell lines were treated with PSPEI polymer at different concentration. Ki-Hyun Cho et al., has shown that PSPEI polyplex had reduced cytotoxicity compared to bPEI, because PSPEI polymer are biodegradable whereas bPEI are non-biodegradable polymers [33]. In Figure 3a, in both cell lines, PSPEI polymer displayed no sign of reduction in the cell viability. Also, immature BMDCs treated with PSPEI-PAA nanocomplex at different ratios were highly viable and represented no significant toxicity (Figure 3b). Therefore, PSPEI-PAA nanocomplex can be acknowledged as a safe vaccine to be administered for in vitro and in vivo methods.

Dendritic cells have the natural ability to uptake foreign antigen especially in form of proteins, although DCs are in immature state in cancer tumor microenvironment and thus lacks ability to uptake antigen more efficiently. Hence, the intracellular uptake of PSPEI-PAA nanocomplex was analyzed in immature dendritic cells (iDCs) (Figure 4a). Compared to OVA-FITC, PSPEI-PAA has shown enhanced intracellular uptake and it can be recognized through the fluorescent intensity of PSPEI-PAA treated iDCs at 20:1 (*w*/*w*) which was higher than that of OVA-FITC. The osmotically active part of PSPEI and cationic surface charge of PSPEI-PAA has supported in the enhanced intracellular uptake in iDCs [37]. Through flow cytometry analysis, it was confirmed that the percentage of OVA-FITC, CD11c+ iDCs were significantly higher (*** *p* < 0.001) in PSPEI-PAA nanocomplex treatment group compared to OVA-FITC treatment alone (Figure 4b).

Previously, Hee Dong Han et al., has demonstrated that the enhanced intracellular uptake of chitosan-poly I:C complex has improved the DC maturation [41]. Hence, we investigated the DC maturation in day6 iDCs treated with PSPEI-PAA nanocomplex for 24 h. Figure 5a,b, shows that PSPEI-PAA nanocomplex treated iDCs have enhanced CD80+/86+ expression compared to PIC-lysate protein mixture treatment. From this, it can be concluded that the presence of poly I:C and lysate protein in the PSPEI-PAA nanocomplex has activated and matured iDCs. Then, the PSPEI-PAA was evaluated for antitumor activity in B16F10 tumor model for 14 days. The antitumor response of PSPEI-PAA was enhanced significantly higher than that of PSPEI-A or antigen along with PIC treated tumor mice (Figure 6a). The treatment has showed no side effects based on the body weight change of the treated tumor mice. PSPEI-PAA containing PIC and protein lysate has been uptaken by the antigen presenting cells like dendritic cells and induced antitumor immune response.

In Figure 7a, the cells isolated from tumor draining lymph node in PSPEI-PAA nanocomplex treated mice have increased expansion of CD11c+CD80+CD86+ matured DCs compared to PBS control mice. Additionally, there was a significant increase in the CD8+ T cell population and reduction in CD4+ FOXP3+ Treg cells in the PSPEI-PAA nanocomplex treated mice tumor compared to PBS control mice tumor (Figure 7b,c). However, there was no significant change in the CD4+ T cell population in all the treatment groups. Previously, it was reported that poly I:C can trigger the antigen specific immune response by directly activating the CD8+ T cells via TLR3 signaling pathway [42]. In addition, it was reported that DCs activated and matured by poly I:C expresses MHC class I and therefore directly initiates the CD8+ cytotoxic T cells [43]. Later, we estimated the antitumor immune response through splenocytes isolated from spleen of treatment tumor mice and later co-cultured with B16F10 cells for 6 h (Figure 8). The LDH release from the PSPEI-PAA splenocytes treated B16F10 cells were augmented significantly with increase in the ratio between spleenocyte to target cancer cells. PSPEI-PAA administration in B16F10 tumor via peritumoral route initiates the antitumor immune response by maturating dendritic cells and in turn activating the cytotoxic T cells through T helper (Th) cells. The activated CTL kills the tumor [44,45]. From this, it can be assessed that the B16F10 lysate protein mediated activation of cytotoxic T cells in the splenocytes has led to killing of the B16F10 melanoma cancer cells.

## 5. Conclusions

Nanovaccine-based tumor immunotherapy is under rapid development and the approach holds tremendous promise. Although past studies have harnessed polymeric nanocarriers as vehicles for delivery of chemo drugs and genes, the studies depicting their ability to coordinately delivery tumor antigens and immune stimulatory molecules to DCs are still immature. Hence, we developed a self-assembled nanocomplex using polysorbitol-polyethylenimine polymer complexed with lysate protein and poly I:C. The PSPEI-PAA has shown better complexation with nucleotide and protein, enhanced intracellular delivery of cargos and effective anti-cancer immune response against melanoma tumor. Based on the flow cytometry data and CTL assay, it was clear that cytotoxic T cells activated in the PSPEI-PAA nanocomplex treated tumor mice were responsible for the prevention of tumor growth. In general, this approach has generated enhanced positive effects compared to the conventional tumor antigen vaccination, in regards with enhanced augmentation of CD8+ T-cell responses against the tumor. Overall, the currents results have promised that PSPEI-PAA nanocomplex could be an efficient nano-based vaccine for future clinical studies.

## References

[1] Siegel R.L., Miller K.D., Jemal A. (2018). Cancer statistics, 2018. CA Cancer J. Clin..

[2] Sanlorenzo M., Vujic I., Posch C., Dajee A., Yen A., Kim S., Ashworth M., Rosenblum M.D., Algazi A., Osella-Abate S. (2014). Melanoma immunotherapy. Cancer Biol. Ther..

[3] Ozao-Choy J., Lee D.J., Faries M.B. (2014). Melanoma vaccines: Mixed past, promising future. Surg. Clin. N. Am..

[4] Yushak M.L., Chiang V.L., Kluger H.M. (2015). Clinical trials in melanoma patients with brain metastases. Pigment Cell Melanoma Res..

[5] Goldberg J.L., Sondel P.M. (2015). Enhancing cancer immunotherapy via activation of innate immunity. Semin. Oncol..

[6] Takakura K., Kajihara M., Ito Z., Ohkusa T., Gong J., Koido S. (2015). Dendritic-tumor fusion cells in cancer immunotherapy. Discov. Med..

[7] Maus M.V., Fraietta J.A., Levine B.L., Kalos M., Zhao Y., June C.H. (2014). Adoptive immunotherapy for cancer or viruses. Annu. Rev. Immunol..

[8] Zhang X., Kang Z., Li Q., Zhang J., Cheng S., Chang H., Wang S., Cao S., Li T., Li J. (2018). Antigen-adjuvant effects of icariin in enhancing tumor-specific immunity in mastocytoma-bearing dba/2j mice. Biomed. Pharmacother..

[9] Seya T., Shime H., Takeda Y., Tatematsu M., Takashima K., Matsumoto M. (2015). Adjuvant for vaccine immunotherapy of cancer--focusing on toll-like receptor 2 and 3 agonists for safely enhancing antitumor immunity. Cancer Sci..

[10] Schijns V., Tartour E., Michalek J., Stathopoulos A., Dobrovolskiene N.T., Strioga M.M. (2014). Immune adjuvants as critical guides directing immunity triggered by therapeutic cancer vaccines. Cytotherapy.

[11] Yuan S., Shi C., Liu L., Han W. (2010). Muc1-based recombinant bacillus calmette-guerin vaccines as candidates for breast cancer immunotherapy. Expert Opin. Biol. Ther..

[12] O’Hagan D.T., Friedland L.R., Hanon E., Didierlaurent A.M. (2017). Towards an evidence based approach for the development of adjuvanted vaccines. Curr. Opin. Immunol..

[13] Bowen W.S., Svrivastava A.K., Batra L., Barsoumian H., Shirwan H. (2018). Current challenges for cancer vaccine adjuvant development. Expert Rev. Vaccines.

[14] Shao K., Singha S., Clemente-Casares X., Tsai S., Yang Y., Santamaria P. (2015). Nanoparticle-based immunotherapy for cancer. ACS Nano.

[15] Zang X., Zhao X., Hu H., Qiao M., Deng Y., Chen D. (2017). Nanoparticles for tumor immunotherapy. Eur. J. Pharm. Biopharm..

[16] Kapadia C.H., Perry J.L., Tian S., Luft J.C., DeSimone J.M. (2015). Nanoparticulate immunotherapy for cancer. J. Control. Release.

[17] Song W., Musetti S.N., Huang L. (2017). Nanomaterials for cancer immunotherapy. Biomaterials.

[18] Saleh T., Shojaosadati S.A. (2016). Multifunctional nanoparticles for cancer immunotherapy. Hum. Vaccinnes Immunother..

[19] Fang R.H., Kroll A.V., Zhang L. (2015). Nanoparticle-based manipulation of antigen-presenting cells for cancer immunotherapy. Small.

[20] Firdous J., Islam M.A., Park S.M., Cheon I.S., Shim B.S., Yoon H.S., Song M., Chang J., Choi Y.J., Park Y.M. (2014). Induction of long-term immunity against respiratory syncytial virus glycoprotein by an osmotic polymeric nanocarrier. Acta Biomater..

[21] Nguyen K.C., Muthiah M., Islam M.A., Kalash R.S., Cho C.S., Park H., Lee I.K., Kim H.J., Park I.K., Cho K.A. (2014). Selective transfection with osmotically active sorbitol modified pei nanoparticles for enhanced anti-cancer gene therapy. Colloids Surf. B Biointerfaces.

[22] Lu H. (2014). Tlr agonists for cancer immunotherapy: Tipping the balance between the immune stimulatory and inhibitory effects. Front. Immunol..

[23] Stier S., Maletzki C., Klier U., Linnebacher M. (2013). Combinations of TLR ligands: A promising approach in cancer immunotherapy. Clin. Dev. Immunol..

[24] Ayari C., Besancon M., Bergeron A., LaRue H., Bussieres V., Fradet Y. (2016). Poly(I:C) potentiates bacillus calmette-guerin immunotherapy for bladder cancer. Cancer Immunol. Immunother..

[25] Forghani P., Waller E.K. (2015). Poly (I:C) modulates the immunosuppressive activity of myeloid-derived suppressor cells in a murine model of breast cancer. Breast Cancer Res. Treat..

[26] Qu J., Hou Z., Han Q., Zhang C., Tian Z., Zhang J. (2013). Poly(i:C) exhibits an anti-cancer effect in human gastric adenocarcinoma cells which is dependent on rlrs. Int. Immunopharmacol..

[27] Gupta S.K., Yadav P.K., Tiwari A.K., Gandham R.K., Sahoo A.P. (2016). Poly (I:C) enhances the anti-tumor activity of canine parvovirus ns1 protein by inducing a potent anti-tumor immune response. Tumour. Biol..

[28] Salazar A.M., Erlich R.B., Mark A., Bhardwaj N., Herberman R.B. (2014). Therapeutic in situ autovaccination against solid cancers with intratumoral poly-iclc: Case report, hypothesis, and clinical trial. Cancer Immunol. Res..

[29] Pitcovski J., Shahar E., Aizenshtein E., Gorodetsky R. (2017). Melanoma antigens and related immunological markers. Crit. Rev. Oncol. Hematol..

[30] Andrews M.C., Woods K., Cebon J., Behren A. (2014). Evolving role of tumor antigens for future melanoma therapies. Future Oncol..

[31] Pichon C., Midoux P. (2013). Mannosylated and histidylated lpr technology for vaccination with tumor antigen mrna. Methods Mol. Biol..

[32] Liu L.N., Shivakumar R., Allen C., Fratantoni J.C. (2008). Delivery of whole tumor lysate into dendritic cells for cancer vaccination. Methods Mol. Biol..

[33] Cho K.-H., Singh B., Maharjan S., Jang Y., Choi Y.-J., Cho C.-S. (2017). Local delivery of ctgf sirna with poly(sorbitol-co-pei) reduces scar contraction in cutaneous wound healing. Tissue Eng. Regen. Med..

[34] Luu Q.P., Shin J.Y., Kim Y.K., Islam M.A., Kang S.K., Cho M.H., Choi Y.J., Cho C.S. (2012). High gene transfer by the osmotic polysorbitol-mediated transporter through the selective caveolae endocytic pathway. Mol. Pharm..

[35] Schaffert D., Kiss M., Rodl W., Shir A., Levitzki A., Ogris M., Wagner E. (2011). Poly(I:C)-mediated tumor growth suppression in egf-receptor overexpressing tumors using egf-polyethylene glycol-linear polyethylenimine as carrier. Pharm. Res..

[36] Syga M.I., Nicoli E., Kohler E., Shastri V.P. (2016). Albumin incorporation in polyethylenimine-DNA polyplexes influences transfection efficiency. Biomacromolecules.

[37] Cho W.Y., Hong S.H., Singh B., Islam M.A., Lee S., Lee A.Y., Gankhuyag N., Kim J.E., Yu K.N., Kim K.H. (2015). Suppression of tumor growth in lung cancer xenograft model mice by poly(sorbitol-co-pei)-mediated delivery of osteopontin sirna. Eur. J. Pharm. Biopharm..

[38] Chou M.J., Yu H.Y., Hsia J.C., Chen Y.H., Hung T.T., Chao H.M., Chern E., Huang Y.Y. (2018). Highly efficient intracellular protein delivery by cationic polyethyleneimine-modified gelatin nanoparticles. Materials.

[39] Menon J.U., Ravikumar P., Pise A., Gyawali D., Hsia C.C., Nguyen K.T. (2014). Polymeric nanoparticles for pulmonary protein and DNA delivery. Acta Biomater..

[40] Kafil V., Omidi Y. (2011). Cytotoxic impacts of linear and branched polyethylenimine nanostructures in a431 cells. Bioimpacts.

[41] Han H.D., Byeon Y., Jang J.H., Jeon H.N., Kim G.H., Kim M.G., Pack C.G., Kang T.H., Jung I.D., Lim Y.T. (2016). In vivo stepwise immunomodulation using chitosan nanoparticles as a platform nanotechnology for cancer immunotherapy. Sci. Rep..

[42] Salem M.L., Diaz-Montero C.M., El-Naggar S.A., Chen Y., Moussa O., Cole D.J. (2009). The tlr3 agonist poly(I:C) targets cd8+ t cells and augments their antigen-specific responses upon their adoptive transfer into naive recipient mice. Vaccine.

[43] Pulko V., Liu X., Krco C.J., Harris K.J., Frigola X., Kwon E.D., Dong H. (2009). Tlr3-stimulated dendritic cells up-regulate b7-h1 expression and influence the magnitude of cd8 t cell responses to tumor vaccination. J. Immunol..

[44] Chandran S.S., Paria B.C., Srivastava A.K., Rothermel L.D., Stephens D.J., Kammula U.S. (2015). Tumor-specific effector cd8+ t cells that can establish immunological memory in humans after adoptive transfer are marked by expression of il7 receptor and c-myc. Cancer Res..

[45] Sckisel G.D., Mirsoian A., Minnar C.M., Crittenden M., Curti B., Chen J.Q., Blazar B.R., Borowsky A.D., Monjazeb A.M., Murphy W.J. (2017). Differential phenotypes of memory cd4 and cd8 t cells in the spleen and peripheral tissues following immunostimulatory therapy. J. Immunother. Cancer.

